# Supplementary material for: Premorbid performances determine the deleterious effects of nigrostriatal degeneration and pramipexole on behavioural flexibility
Source: NPJ Parkinsons Dis. 2023 Mar 1;9:31. doi: 10.1038/s41531-023-00475-3 (PMC9977907; doi:10.1038/s41531-023-00475-3)
Supplement: Supplementary file 1 — supplementary figure 1 [file 41531_2023_475_MOESM1_ESM.pdf]

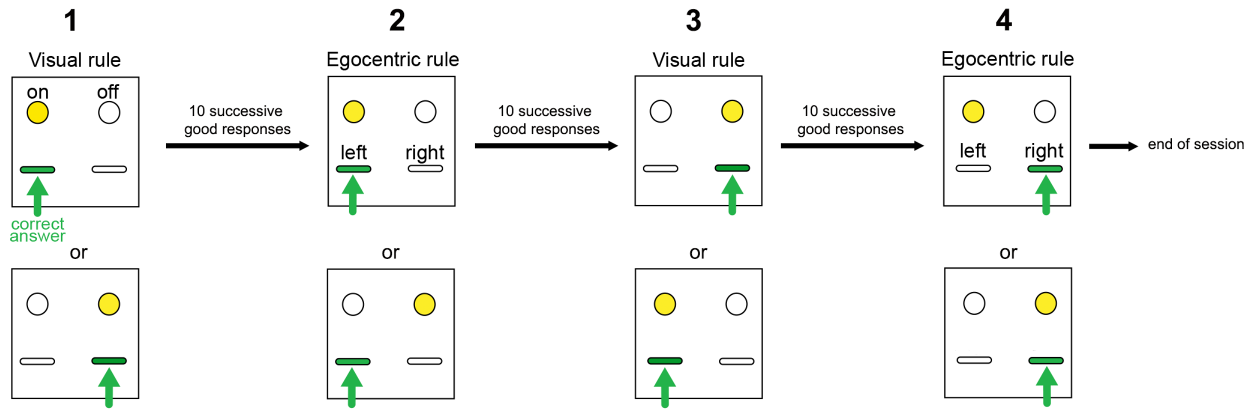

Supplementary Figure 1 : *organization of the set-shifting task*

The task is composed of 4 alternating visual (1, 3) and egocentric rules (2, 4). In the visual rule (1, 3), the position of the rewarded lever (green) is indicated by the position of the light cue (yellow) above it. In this rule, the position of the rewarded lever can randomly alternate between left and right (together with the light cue) but cannot be at the same position for more than two consecutive trials. In the egocentric rule (2, 4), the position of the rewarded lever (green) is fixed at the beginning of the rule (either left as shown in 2, or right as shown in 4), whereas the position of the light cue (yellow) is random. Each rule is completed when 10 successive correct responses have been made, and the task then progresses to the next rule. The session ends after 4 rules have been completed (or after 45 minutes if less than four rules have been completed). The sequence of rules is defined for each rat in a pseudo-random order during the first training session: the first rule is randomly selected and then the task progresses in a logical order (e.g. 1-2-3-4, 2-3-4-1, 3-4-1-2 or 4-1-2-3).
